# Data Ownership and Privacy in Dairy Farming: Insights from U.S. and Global Perspectives

**DOI:** 10.3390/ani15040524

**Published:** 2025-02-12

**Authors:** Richard Barton, Javier Burchard, Victor E. Cabrera, David Cook, Walter Cooley, Roger Cue, Liliana Fadul, Jay Mattison, Amit Saha

**Affiliations:** 1 Retired USDA-NASS-ERS, Stoughton, WI 53589, USA; barton.ra@outlook.com; 2 Council of Dairy Cattle Breeding, Bowie, MD 20716, USA; javier.burchard@uscdcb.com; 3 Department of Animal and Dairy Sciences, University of Wisconsin–Madison, Madison, WI 53706, USA; 4 Bovisync, Fond du Lac, WI 54935, USA; dcook@bovisync.com; 5 AgProud, Jerome, ID 83338, USA; walt.cooley@agproud.com; 6 Department of Animal Science, McGill University, Sainte-Anne-de-Bellevue, QC H9X 3V9, Canada; roger.cue@mcgill.ca; 7 Lactanet, Sainte-Anne-de-Bellevue, QC H9X 0B9, Canada; lfadul@lactanet.ca; 8 National Dairy Herd Information Association, Verona, WI 53593, USA; jmattison@requestltd.com; 9 FoGS Global Research and Consultancy Center, Ahmedabad 380051, India; fogsconsulting@outlook.com

**Keywords:** data ownership, privacy in dairy industry, technological integration, equitable value distribution, global data standards

## Abstract

In the evolving landscape of dairy farming, data ownership and privacy have become critical issues. This commentary paper delves into the complexities and implications of data management in the dairy industry, informed by insights from a multidisciplinary group of stakeholders. While the authors bring a U.S. perspective, the challenges discussed are globally relevant, given the dominant role of multinational corporations in shaping data practices across regions. The discussions, conducted through structured meetings and iterative online exchanges, emphasized the pressing legal and ethical concerns, the role of technology, and the necessity of developing comprehensive guidelines to safeguard data integrity and benefit all stakeholders. Key points include the global nature of data protection regulations, the potential of blockchain and IoT devices to enhance transparency, and the importance of equitable value distribution. By fostering an environment of transparency and fairness, the dairy industry can harness the power of data to drive innovation and sustainability while protecting the rights of those who generate it. This paper provides a pathway to address these challenges by raising awareness and proposing general actions for the industry’s future.

## Simple Summary

The dairy industry depends on data to improve productivity and sustainability. However, this raises concerns about who owns the data and how it is used. In this paper, a group of experts discussed the challenges of data ownership and privacy in dairy farming. The group emphasized the need for clear rules to protect farmers and ensure data are used ethically. They also explored how new technologies like sensors, connected devices, and the blockchain can improve data management. The group proposed general actions to encourage fairness and transparency in data sharing while safeguarding the rights of farmers. This paper aims to raise awareness of these issues and guide future solutions.

## 1. Introduction

The dairy industry is increasingly reliant on data-driven technologies to enhance productivity and efficiency [1]. This dependency raises significant concerns regarding data ownership and privacy. As data are collected, processed, and utilized by various entities, it is imperative to establish clear guidelines and policies to protect the interests of all stakeholders, particularly those directly involved in generating and managing data on the farm [2]. For example, similar data ownership challenges are observed in crop farming, where precision agriculture tools generate vast amounts of data on soil health, crop yields, and environmental conditions. Like dairy farmers, crop farmers often face uncertainties about who controls these data once they are shared with agribusinesses or technology providers. The situation parallels the dairy industry’s challenges, where data from IoT (Internet of things) devices or herd management systems must be managed transparently to ensure farmer autonomy and equitable benefits. These examples underscore the urgent need for comprehensive frameworks across agricultural sectors to address data ownership and privacy concerns.

The dairy industry is increasingly reliant on data-driven technologies to enhance productivity and efficiency. However, this dependency raises significant concerns regarding data ownership and privacy. As data are collected, processed, and utilized by various entities, it is imperative to establish clear guidelines and policies to protect the interests of all stakeholders, particularly the farmers who generate these data.

Additionally, the global nature of the dairy industry necessitates standardized legal frameworks for data management, highlighting discrepancies in data protection laws such as the General Data Protection Regulation (GDPR) in Europe and varying regulations in countries like India and the USA [3]. However, these frameworks are often insufficient for the global dairy industry, as they primarily address individual privacy rather than the collective and complex nature of agricultural data. For instance, GDPR does not account for the intricacies of ownership and the use of data generated on farms by IoT devices or herd management systems. Furthermore, the fragmented regulatory environment across regions like the USA, India, and Europe exacerbates these challenges, making it difficult to establish consistent data management practices on a global scale.

The integration of advanced technologies, such as IoT devices and the blockchain, presents both opportunities and challenges. These technologies can enhance data tracking and transparency but also raise questions about data security and farmer consent. Addressing these challenges requires a multifaceted approach that includes equitable frameworks, robust data protection, and global compliance measures to navigate the complexities of data ownership and privacy in dairy farming [4].

By fostering an environment of transparency and fairness, the dairy industry can harness the power of data to drive innovation and sustainability while safeguarding the rights of those who generate it. This paper aims to provide a comprehensive overview of the discussions held by a multidisciplinary group of stakeholders, focusing on challenges related to data ownership, privacy, and usage in dairy farming. While grounded in the U.S. context, the insights and recommendations presented are broadly applicable to global dairy farming systems due to the universal relevance of these issues.

## 2. Materials and Methods

We utilized a subgroup of the Dairy Brain Coordinated Innovation Network (CIN; [5,6]) an initiative led by the University of Wisconsin–Madison to address data management challenges in dairy farming. The CIN fosters collaboration among farmers, researchers, industry professionals, and technology developers to enhance data integration, improve decision-making, and promote sustainable practices. This multidisciplinary group convened to explore critical issues of data ownership and privacy, aiming to develop a commentary paper that provides guidance and insights for the industry. The primary objectives were to assess the current state of data ownership and privacy, identify key challenges, and propose potential solutions. The discussions were centered on data generated on dairy farms, encompassing a wide range of data types, including production, health, management, and environmental metrics.

The discussions that informed this commentary paper were held through two structured meetings, supplemented by extensive online and email exchanges between the period June and December 2024. The participants in these meetings were the same as the authors of this manuscript, representing diverse expertise in dairy farming, data management, academia, and consultancy. The stakeholder group was intentionally composed to represent perspectives critical to the dairy data ecosystem, including academic researchers, consultants, and technology developers, ensuring a holistic approach to the issues discussed.

The meetings were organized around key themes to ensure focused and productive discussions. Meeting 1 addressed the foundational concepts of data ownership, privacy, and the ethical considerations associated with their use in the dairy industry. Participants also explored global data protection frameworks and identified gaps relevant to the dairy sector. Meeting 2 focused on practical solutions and recommendations, including the role of advanced technologies like IoT and the blockchain, the feasibility of data monetization, and strategies for equitable value distribution.

IoT devices are physical devices embedded with sensors, software, and connectivity that allow them to collect and exchange data. Examples in dairy farming include sensors that monitor cow health, milk yield, and environmental conditions, providing real-time data to support decision-making and farm management. Blockchain refers to a decentralized and distributed ledger technology designed to record transactions securely and transparently. In the context of the dairy industry, the blockchain has been proposed as a tool to enhance traceability, monitor data usage, and ensure the equitable distribution of benefits derived from data.

The discussions were designed to be high level, focusing on identifying critical challenges and proposing general actions to address them. The aim was not to quantify stakeholder perspectives or measure agreement on specific solutions but rather to foster a collaborative dialog and raise awareness of the complexities surrounding data ownership and privacy. This approach serves as a foundational step, with the intention of guiding future research that can incorporate quantitative or qualitative analyses to explore stakeholder preferences and solutions in greater depth.

Collaboration was facilitated using a combination of video conferencing platforms and shared online documents. AI-assisted tools were employed for notetaking, ensuring the accurate and efficient documentation of the discussions. The iterative process of online communications allowed the group to refine and synthesize key findings and recommendations collectively. The approach also emphasized the importance of adherence to a code of conduct, ensuring that all participants felt secure in sharing their insights and concerns.

While the multidisciplinary group included researchers, consultants, and industry leaders with diverse expertise, certain limitations should be acknowledged. The geographic diversity of participants was limited, with most participants representing North America. This may have influenced the discussions by prioritizing challenges and perspectives more common to these regions. Additionally, while the group included industry leaders with direct links to farmers, the limited representation of active farmers in the discussions may have constrained the depth of firsthand insights into on-farm challenges. These limitations highlight the need for broader, more geographically diverse engagement and greater farmer involvement in future discussions to ensure that proposed solutions are universally applicable and grounded in practical realities.

## 3. Results

**Figure 1:**
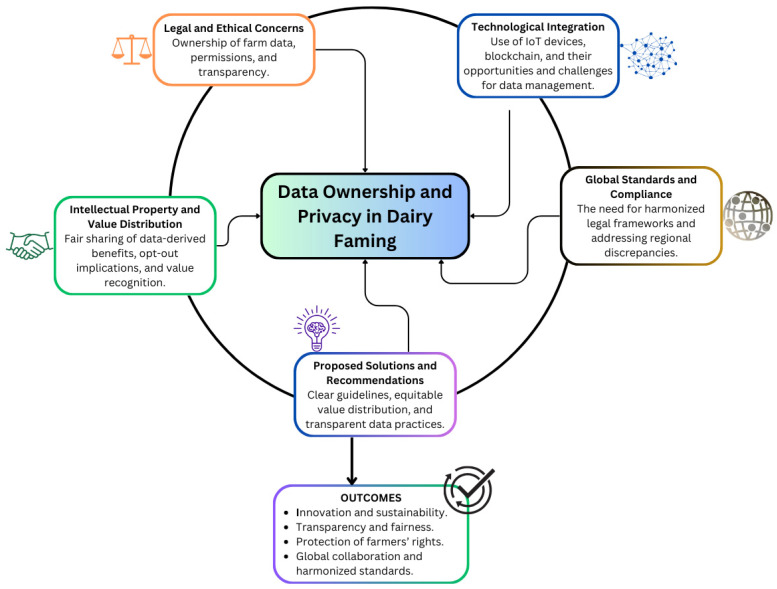
Key themes and solutions for addressing data ownership and privacy challenges in dairy farming. The diagram highlights the central focus on data ownership and privacy and their relationship with legal and ethical concerns, intellectual property, technological advancements, global standards, and proposed solutions. The outcomes emphasize transparency, sustainability, the protection of farmers’ rights, and global collaboration.

The overarching themes and outcomes identified in this paper are summarized in Figure 1; they emerged from the structured discussions and subsequent analysis. These themes build upon the discussion segments outlined earlier, which focused on foundational concepts, global frameworks, technological advancements, and value distribution. Together, they provide a comprehensive framework to address the critical challenges facing the dairy industry. Figure 1 summarizes these relationships, illustrating the major challenges, proposed solutions, and broader impacts.

### 3.1. Key Challenges in Data Ownership and Governance

#### 3.1.1. Legal and Ethical Concerns

The legal and ethical landscape of data ownership in the dairy industry is multifaceted and complex. The consensus among stakeholders is that data generated on farms inherently belong to the farmers. This perspective is critical as it parallels data privacy issues faced by tech giants like Meta and Google, emphasizing the need for explicit permissions and transparent communication regarding data usage [7].

A significant concern arises when farm data are used without explicit permission, leading to potential misuse and ethical dilemmas. The complexity of data ownership intensifies when external entities process and utilize these data. It is crucial to have clear and explicit permissions and communication regarding the use and implications of farm data.

Farmers’ perceptions of data ownership issues reflect a combination of optimism and concern. While recognizing the potential of data sharing to drive innovation and improve farm management, farmers often express unease regarding their lack of control over data once shared with external entities. This sentiment is particularly strong when data are used for purposes beyond their original intent, raising questions about fairness and transparency. Many farmers advocate for clear agreements that outline data usage rights and ensure equitable compensation for contributions, which are crucial for fostering trust and broader participation in data-driven initiatives.

Defining the ownership of processed data and managing the intellectual property derived from such data are also critical issues. The current discussions highlight the necessity for a comprehensive policy framework that addresses these concerns, ensuring that farmers retain ownership and control over their data throughout the data’s lifecycle. This approach would involve creating policies that favor farmers, enabling them to manage and control changes related to their data.

Furthermore, the issue of terms and conditions associated with technology and software used in farming was discussed. Farmers often accept these terms without fully understanding their implications, which can lead to the loss of control over their data once they leave the farm. Additionally, farmers may not fully recognize the value of their individual data, particularly when they become part of a larger benchmarking system that benefits the broader industry. This lack of awareness underscores the need for more readable and concise terms and conditions, ensuring farmers are not only informed about what they are agreeing to but also empowered to understand how their data contribute to aggregated systems and the potential returns they generate. Clear and accessible agreements are essential to promote transparency and build trust between farmers and technology providers.

Finally, the ethical and legal implications of data collected by agri-technology providers underscore the need for transparency and fair compensation for farmers. While the discussions did not explicitly use the FAIR (findable, accessible, interoperable, and reusable) concept, its principles resonate strongly with the group’s emphasis on transparency, equitable access, and the responsible use of data. Incorporating FAIR principles into the dairy industry’s data management frameworks could further enhance interoperability and reusability, ensuring that all stakeholders, particularly farmers, derive maximum value from their data. Robust data protection measures must be implemented to safeguard against misuse and unauthorized access, ensuring the benefits of data collection and processing are equitably distributed among all stakeholders, especially the farmers. The involvement of milking companies and Farm Management Information System (FMIS) providers introduces additional complexity to the landscape of data ownership. These entities often harvest data from milking parlors through integrated systems and direct connections to FMIS platforms. Such practices, typically governed by contractual agreements, facilitate seamless data flow and provide valuable insights for farm management. However, they also raise significant questions about ownership and control. Farmers may lack clarity regarding how their data are being used, who has access to them, and whether they are adequately compensated for their use in developing advanced management tools or analytics. Establishing transparent agreements and clear ownership definitions between farmers, milking companies, and FMIS providers is essential to addressing these challenges and fostering equitable practices across the dairy data ecosystem.

The potential integration of environmental and health-related data into broader datasets introduces significant sensitivities, particularly when such data are accessed or utilized by national agencies or ministries for policymaking. The implications of these outcomes for the farming community can be profound, influencing both trust in data-sharing frameworks and the acceptance of resulting policies. Ensuring transparency about how aggregated data will be used and engaging stakeholders early in the process are critical to mitigating these concerns. Farmers must be assured that their data contributions are used responsibly and transparently, with governance mechanisms in place to prevent unintended consequences such as excessive regulatory requirements or the mischaracterizations of their practices. While farmers may not have a direct influence over the content of regulations, fostering transparency and collaboration between stakeholders can help ensure that data are used in ways that support equitable and practical outcomes.

#### 3.1.2. Intellectual Property and Value Distribution

The critical discussion points are centered around the intellectual property (IP) rights associated with data-derived products. Emphasizing the need to equitably share the benefits derived from data, it was acknowledged that farmers’ contributions must be recognized and fairly compensated. One proposal was for the development of a framework that clearly defines ownership at different stages of data processing—raw, intermediate, and processed data. Raw data refer to the unprocessed information collected directly from sources such as sensors, milking parlors, or farm management systems. This stage includes primary measurements like milk weights, feed intakes, and environmental parameters. Intermediate data represent information that has undergone initial processing, such as summaries, averages, or basic calculations derived from raw data. Processed data refer to highly refined outputs, such as predictive models, benchmarking reports, or decision-support tools generated through advanced analytics or machine learning.

Beyond categorizing data as raw, intermediate, or processed, it is crucial to consider their transformation into actionable insights. This progression—from data to information, contextual knowledge, and decision-making—adds significant value. For instance, raw milk production data gain utility when combined with environmental and historical benchmarks, enabling better farm management decisions. Frameworks should recognize and address value distribution across this chain, ensuring transparency and fair compensation as data evolve into actionable knowledge. IP considerations vary at each stage. For raw data, ownership typically lies with the entity that generates them, such as the farmer or farm organization. However, when intermediate and processed data are created, IP rights often extend to the organizations or entities responsible for adding value through processing or analytics. This progression introduces complexities in defining ownership and control, emphasizing the need for clear policies to delineate rights at each stage of the data’s lifecycle. These policies should ensure that farmers retain control over raw data while benefiting equitably from the value derived in subsequent stages.

It is essential to outline the scope and content of documents related to data ownership and privacy. Although the industry generally agrees that farmers own the data, this ownership often becomes less concrete once the data leave the farm or a copy of them is made. Participants noted the necessity for more control mechanisms to ensure that farmers’ rights over their data are preserved. The conclusion that data ownership primarily resides with farmers was unanimously supported by the participants. However, there were differing opinions on the mechanisms required to ensure farmers maintain control over processed data, reflecting the complexity of balancing ownership rights and technological advancements.

Clear definitions and frameworks for data ownership, control, and access are crucial. The discussion highlighted differences in data privacy concerns based on herd sizes and the potential implications of business intelligence derived from large-scale data collection. Data processing centers have evolved from monthly herd recordings to more continuous and detailed data capture, such as daily milk weights and sensor data.

Furthermore, participants stressed the global dimension of data ownership, focusing on the need for standardized laws and compliance measures. It was proposed to classify farm data into different levels—personal, public, and critical. The use of digital technologies like the blockchain was suggested to enhance transparency, verify the origin, and monitor the routing of data. While the blockchain facilitates traceability, its potential to ensure benefits for farmers depends on how it is integrated into broader frameworks for equitable value distribution. Building farmers’ awareness and skills in using digital technologies for data management was deemed crucial.

The intrinsic value of data, particularly in its ability to generate actionable insights and decision-making tools, drives the questions surrounding ownership. Transparency about how producer data are used and whether producers can opt out of specific data flows remains critical for maintaining trust and fairness in the industry. For example, producers who choose not to allow their data to flow to collective entities such as CDCB or breed organizations might forgo access to specific derived services, like estimated breeding values (EBVs). In cases where data-derived models or tools are developed by producer-owned organizations, the idea of quid pro quo has merit. Producers who opt out of contributing data could potentially be charged higher fees for the use of tools developed through collective data efforts [8]. This approach ensures fairness by recognizing the contribution of data-sharing participants while still providing access to the tools for non-contributors. To provide concrete examples of frameworks facilitating data accessibility and sharing, JOIN DATA (https://join-data.nl; accessed 23 January 2025) and DJUSTCONNECT (https://www.djustconnect.be; accessed 23 January 2025) can serve as models. JOIN DATA, developed in the Netherlands, is a platform designed to enable farmers and stakeholders to share data securely through permission-based systems. Similarly, DJUSTCONNECT, primarily utilized in Belgium, focuses on fostering trust and transparency among stakeholders by creating standardized data-sharing agreements and tools. These frameworks highlight how collaborative platforms can address data ownership and privacy challenges while ensuring equitable value distribution among stakeholders. However, these initiatives were not part of our discussions. Future work would benefit from exploring these models and their broader applications.

The group acknowledged that while technological advancements can provide significant benefits, they also introduce complexities in defining and managing IP rights [9]. Therefore, establishing clear guidelines for IP rights associated with data-derived products and ensuring transparency in how data are used and shared among different entities were considered essential steps towards fair and ethical data management practices in the dairy industry.

#### 3.1.3. Technological Advances and Data Management

The integration of advanced technologies, such as IoT (Internet of Things) devices and the blockchain, presents both opportunities and challenges in the dairy industry. These technologies can enhance data tracking and transparency but also raise questions about data security and farmer consent. Blockchain technology, in particular, has been highlighted for its potential to provide transparency, traceability, and secure routing of data, which can facilitate equitable benefit distribution to farmers. The ethical and legal implications of data collected by agri-technology providers necessitate transparency and fair compensation for farmers.

Advances in data collection technologies were emphasized, highlighting the increasing frequency and volume of data being collected. These data, while beneficial for improving farm management, raise significant concerns about data ownership and confidentiality. Technological advancements in data collection and management must ensure that all stakeholders, particularly those directly involved in generating and managing data on the farm, share the benefits of these technologies equitably. Robust data protection measures are needed to safeguard against misuse and unauthorized access.

The potential of digital technologies like the blockchain to enhance transparency and traceability in data management was discussed. These technologies can ensure farmers receive fair compensation for the use of their data and that any technology developed using farm data benefits the farmers. The challenges and opportunities presented through the increasing use of sensor data and IoT devices in dairy farming were also covered. Additionally, FMIS and milking parlors have historically served as primary sources of data in the dairy industry, with strong integration between the two systems. With the advent of the Internet and remote connectivity, IoT technologies have expanded the scope of data collection, complementing these established systems. Clear guidelines on data ownership and access, particularly in relation to processed and intermediate data, are necessary. Ensuring the ethical use of data collected from large herds as well as preventing any disadvantage to smaller farms is crucial.

The economic and environmental impact of implementing technologies like the blockchain was also considered. Although the blockchain can provide significant benefits in terms of data traceability and security, it is expensive to implement and has a considerable environmental footprint due to its high energy consumption. Energy-intensive systems like proof of work (PoW) contribute to high electricity consumption and carbon emissions, which is problematic for the dairy industry’s sustainability goals. To mitigate these issues, energy-efficient alternatives such as proof of stake (PoS), permissioned blockchains, or hybrid systems that combine the blockchain with centralized databases can be considered. Other secure technologies like distributed ledgers and federated learning systems also offer potential solutions with lower environmental impacts. Additionally, powering blockchain operations with renewable energy sources can further reduce their carbon footprint, aligning technological advancements with the industry’s commitment to sustainability.

Emerging trends such as artificial intelligence (AI) and predictive analytics amplify the importance of addressing data ownership and privacy in the dairy industry. These technologies depend on data collected from farms to optimize decision-making, enhance efficiency, and predict trends, raising concerns about control, consent, and equitable benefit distribution. As raw data are transformed into actionable insights, questions emerge about ownership and intellectual property rights. Transparent data-sharing agreements and robust frameworks are essential to ensure that the benefits derived from these technologies are distributed equitably among all stakeholders while addressing the ethical, legal, and social implications of these advancements.

Data utility for commercial, public, and private decision-making was also highlighted as a critical aspect of technological advancements. The role of AI development in global data accessibility and its secure access need to be streamlined to benefit all stakeholders. A sustainable approach to data generation, distribution, and utility requires priority focus at both granular and regional global levels. This holistic approach ensures that data-driven technologies enhance decision-making processes while maintaining sustainability and ethical standards in the dairy industry.

#### 3.1.4. Global Compliance and Standards

The global nature of the dairy industry necessitates standardized legal frameworks for data management. Discrepancies in data protection laws, such as GDPR (General Data Protection Regulation) in Europe [3] and varying regulations in countries like India and the USA, were highlighted. Establishing global compliance standards is essential to protect data integrity and ensure fair use. The variability in the interpretation of global data protection regulations like GDPR underscores the need for harmonized compliance standards. While this paper touches on these differences, a detailed comparative analysis of regional regulatory frameworks was beyond the scope of our discussions.

Organizations like the International Committee for Animal Recording (ICAR; https://www.icar.org; accessed on 3 January 2025) and AgGateway (https://aggateway.org; accessed on 3 January 2025) play pivotal roles in fostering global standardization and interoperability in data management. ICAR provides leadership in standardization and certification in animal data recording and management, ensuring that data collected across diverse regions and technologies can be utilized effectively and consistently. This work promotes trust and transparency among stakeholders. Similarly, AgGateway advances data interoperability and collaboration across the agricultural industry by creating common standards and facilitating seamless data exchange. These organizations represent key contributors to building a robust, secure, and equitable data management ecosystem in the dairy sector.

Differences in data protection regulations across various regions pose challenges for establishing uniform data management practices. A collaborative approach is needed to develop global compliance standards that protect data integrity and ensure fair use. The cooperative nature of the dairy industry in Canada, where data management practices are more standardized and industry-driven, was highlighted. In contrast, the fragmented nature of the dairy industry in the USA, with varying regulations and standards, presents additional challenges for developing uniform data management practices.

In India, the landscape of data management in dairy farming is characterized by significant variability in farm sizes and levels of technological adoption. While large farms are increasingly adopting digital technologies for data collection, small farms often rely on traditional methods, such as handwritten logs, basic spreadsheets, or standalone devices that are not integrated into broader data systems. These examples were chosen based on the expertise and regional focus of the group members, serving to illustrate variability rather than to provide a comprehensive representation of global practices. The lack of standardized legal frameworks for data protection and the need for greater transparency and farmer awareness were emphasized.

A crucial point discussed was the need for policies that favor farmers, ensuring they retain ownership and control over their data. This involves developing policies that are not only effective but also practical and financially feasible. The cooperative approach to policymaking, as seen in some regions of Europe, can serve as a model, despite the challenges and costs involved. Beyond individual farmers, their collective roles within cooperatives and branch organizations hold immense potential to strengthen bargaining power and advocate for equitable data ownership frameworks.

There is also an emphasis on the need for more readable and understandable terms and conditions for technology use in the dairy industry. Current practices often present lengthy and complex documents that users, including farmers, may not fully understand before agreeing to them. Simplifying these terms could enhance transparency and ensure that users are fully aware of their rights and obligations.

Emerging concepts such as data monetization and the establishment of data spaces have gained traction across industries, including agriculture. These frameworks present opportunities for farmers to derive financial benefits from their data while ensuring secure sharing through standardized platforms. Recent advancements highlight the potential of creating farmer-centric data cooperatives that prioritize equitable value distribution and secure data handling.

Finally, the potential role of digital technologies, such as the blockchain, in ensuring data traceability and secure access was discussed. Blockchain technology, while still in its nascent stages in the dairy industry, could provide a robust framework for managing data ownership and ensuring that the benefits of data use are fairly distributed among all stakeholders, particularly farmers [10,11].

### 3.2. Proposed Solutions and Recommendations

#### 3.2.1. Developing Comprehensive Guidelines

The primary recommendation is to develop a set of comprehensive guidelines addressing data ownership, privacy, and intellectual property in the dairy industry. These guidelines should include considerations for their applicability at national, regional, and global levels. Given the interconnected nature of the dairy industry and its reliance on global supply chains and technologies, a multi-tiered approach may be most effective. National guidelines can address specific regulatory and cultural contexts, while regional and global frameworks can harmonize practices across borders, ensuring consistency and equitable standards in the use and protection of data:

- Defining Clear Ownership: We must establish clear ownership at different stages of data processing—raw, intermediate, and processed data. This will help delineate responsibilities and rights associated with each data stage;
- Transparent Communication: We must ensure transparent communication about data usage. Farmers and stakeholders should be informed about how their data are being used, who has access to them, and for what purposes;
- Robust Data Protection Measures: We must implement robust data protection measures to safeguard against unauthorized access and misuse. This includes setting up secure data storage and transfer protocols to maintain data integrity and confidentiality;
- Equitable Distribution of Benefits: We must promote the equitable distribution of benefits derived from data. Farmers should receive fair compensation and recognition for their contributions to data generation, ensuring that the value derived from data is shared among all stakeholders;
- Enhanced Farmer Awareness and Training: We must empower farmers with knowledge about their data rights and the implications of data sharing. Initiatives to build farmer awareness and provide training on digital technologies and data management practices are crucial. This will enable farmers to make informed decisions about data sharing and usage. Transparency about the specific uses of producer data is essential. Producers should have a clear understanding of where their data are being used, how they contribute to the development of tools or models, and what opting out might entail in terms of access or pricing for derived services;
- Collaborative Efforts: We must encourage collaboration among stakeholders—farmers, industry players, academics, and policymakers. Creating platforms for ongoing dialog and cooperation can help address emerging issues and refine data management practices related to ownership and privacy;
- Data Control: Guidelines should also address whether producers can control how far their data flow beyond their immediate purpose. For example, a producer who consents to sharing data with DHIA for reporting might not want the data forwarded to other organizations, such as CDCB or breed associations. This aligns with the accessibility principle of the FAIR data framework, emphasizing that access to data should be managed with clear permissions and transparency. Integrating FAIR principles into data-sharing agreements can help ensure producers retain control over their data while facilitating its ethical and efficient use across the industry. Developing policies to support opt-out options while balancing the collective benefits of shared data can provide a framework for fairness and transparency. While participants agreed on the importance of transparency in data usage, varying viewpoints emerged regarding the feasibility of implementing opt-out mechanisms for data-sharing agreements. Some participants expressed concerns about potential inefficiencies, whereas others highlighted the importance of preserving farmer autonomy.

#### 3.2.2. Intellectual Property and Value Distribution

A critical point raised concerns about the intellectual property rights associated with data-derived products. IP encompasses various forms of protection, such as patents, copyrights, trademarks, and trade secrets, each with distinct applications and implications. In this context, the discussion focused on how data ownership interacts with IP principles, particularly in delineating ownership at different stages of data processing—raw, intermediate, and processed data. The necessity of equitably sharing the benefits derived from data by recognizing the significant contributions of farmers was emphasized. One of the primary challenges identified is defining ownership at different stages of data processing—raw, intermediate, and processed data. It is essential to delineate the scope and content of documents related to data ownership and privacy to address these challenges effectively.

Participants highlighted the need for more robust control mechanisms to ensure farmers’ rights over their data, even after they have been processed by third parties. This includes establishing clear guidelines for intellectual property rights associated with data-derived products and ensuring that farmers receive fair compensation for their data.

The complexity of data ownership is exacerbated by the different stages of data processing. For instance, raw data collected by sensors on the farm are transformed through algorithms and calculations into valuable information. Determining whether the processed data still belong to the farmer or become a shared asset is a critical issue that needs to be addressed. Participants suggested that experimental policymaking, such as defining a legal context for a particular case study, could help in understanding and establishing ownership rights across the data processing chain.

To illustrate the complexities of data-sharing agreements, consider the following examples:

1. Farmer-Owned Data Sharing with a Technology Provider: A farmer consents to sharing raw sensor data from their milking parlor with a technology provider in exchange for access to advanced decision-support tools. While the agreement outlines that the raw data remain as the farmer’s property, the processed data generated by the provider—such as predictive health models for the herd—are retained by the provider under intellectual property rights. This arrangement provides clear benefits to both parties but underscores the importance of transparency and explicit terms in data-sharing agreements;
2. Collaborative Data Pooling for Benchmarking: A cooperative of dairy farmers agrees to pool anonymized herd data to create benchmarking tools managed by a producer-owned organization. The cooperative retains ownership of the raw data, while the benchmarking reports are made available to all members at no additional cost. Farmers who opt not to participate in data pooling are still allowed access to the tools but are required to pay a subscription fee. This example highlights how equitable value distribution can be achieved through well-defined data-sharing agreements that incentivize collaboration while respecting individual choices.

The discussion also touched on the need for transparency in how data are used and shared among different entities. It was noted that the current lack of policy regarding the transformation of raw data into processed data allows for speculation and uncertainty. Therefore, a framework for equitable value distribution should be developed, recognizing the contributions of stakeholders who generate the data, such as farmers.

Building farmers’ awareness and skills in using digital technologies for data management was deemed crucial. The group proposed using digital technologies like the blockchain to monitor data usage and ensure that benefits flow back to the farmers. Blockchain technology, with its ability to provide traceability and transparency, could be an effective tool in ensuring that farmers receive proper credit and value for their data contributions.

A proposed framework for equitable value distribution is as follows:

- Establish Clear Guidelines: we must define intellectual property rights at different stages of data processing;
- Ensure Fair Compensation: We must implement mechanisms to ensure farmers receive equitable benefits from their data. This recommendation acknowledges the need to balance fairness with the broader dynamics of the dairy data market, raising questions about whether this market should be regulated or operate as an open system;
- Promote Transparency: we must develop transparent data management practices that clarify how data are used and shared;
- Utilize Digital Technologies: we must employ technologies like the blockchain to trace data usage and ensure proper value distribution;
- Build Awareness and Skills: we must empower farmers with knowledge and skills in digital data management to protect their interests.

#### 3.2.3. Technological Integration

The integration of advanced technologies, such as IoT devices and the blockchain, presents opportunities to enhance data tracking and transparency, though concerns about security and farmer consent remain. Historically, FMIS and milking systems laid the groundwork for dairy data practices, evolving from localized data collection to real-time sharing enabled via IoT. Recent advancements in data platforms and data spaces, such as JOIN DATA and DJUSTCONNECT in Europe, provide secure, permission-based frameworks that enhance data accessibility and interoperability. The blockchain, while ensuring transparency and data provenance, underscores the need for robust guidelines to address both the benefits and challenges of these technologies in the dairy industry.

Beyond high-level potential, IoT and blockchain technologies are now being employed in agriculture to enhance operational efficiency and traceability. For instance, IoT sensors provide real-time monitoring of herd health and environmental conditions, enabling data-driven decision-making. Blockchain systems, on the other hand, are used for secure data sharing in supply chains, ensuring data integrity while facilitating farmer access to decentralized platforms for collective decision-making.

Data spaces have emerged as transformative frameworks for secure, federated data sharing, addressing concerns about data ownership, privacy, and interoperability. These systems enable multiple stakeholders to collaborate within a trusted environment while maintaining sovereignty over their respective data. For example, the International Data Spaces Association (IDSA) has developed a reference architecture model (https://www.internationaldataspaces.org; accessed 23 January 2025) that ensures data sovereignty and facilitates interoperability across industries, including agriculture. Similarly, the European Commission’s guidelines on agricultural data spaces (https://ec.europa.eu; accessed 23 January 2025) emphasize their potential to promote transparency, equitable value distribution, and innovation through standardized protocols and access controls. For the dairy sector, data spaces could provide solutions to unauthorized data use, fostering trust and collaboration among farmers, technology providers, and policymakers. By integrating data spaces into the broader technological landscape, the industry can enhance data governance and drive sustainable advancements in precision agriculture.

While recent developments such as data platforms and data spaces represent promising avenues for advancing data sharing and collaboration, these topics were not explicitly discussed in our group meetings. Future work should explore their implications for data ownership, privacy, and equitable value distribution in dairy farming.

#### 3.2.4. Enhancing Data Utility for Decision-Making

A crucial point discussed was the role of data utility in decision-making processes across commercial, public, and private sectors. By leveraging AI development and machine learning tools, the dairy industry can enhance decision-making at multiple levels—granular, sectoral, regional, and global. Ensuring secure global data accessibility remains a long-term aspiration, requiring robust frameworks for data standardization, security, and producer consent. A sustainable approach to data generation, distribution, and utility is essential to achieving this goal while balancing local and global perspectives. This approach will empower the industry to gain competitive and sustainable insights while safeguarding individual producers’ rights and data integrity.

#### 3.2.5. Addressing the Ethical and Legal Implications

The ethical and legal implications of data collected by agri-technology providers were a major concern. Participants emphasized the need for transparency and fair compensation for farmers. The increasing frequency and volume of data being collected through advanced technologies should be managed to ensure that the benefits of these technologies are equitably shared among all stakeholders, particularly the farmers who generate the data. Robust data protection measures are essential to safeguard against misuse and unauthorized access.

#### 3.2.6. Implementing Blockchain and IoT Technologies

The potential of digital technologies like the blockchain to enhance transparency and traceability in data management was highlighted. Using these technologies can ensure that farmers receive fair compensation for the use of their data and that any technology developed using farm data benefits the farmers. The challenges and opportunities presented through the increasing use of sensor data and IoT devices in dairy farming were also covered. Clear guidelines on data ownership and access, particularly in relation to processed and intermediate data, are necessary. Ensuring the ethical use of data collected from large herds as well as preventing any disadvantage to smaller farms is crucial. Furthermore, for a transparent value chain from farmer to consumer, stakeholders need to form a trustable organization connected through a digital platform between the stakeholders for mapping existing supply management practices.

#### 3.2.7. Economic and Environmental Impacts

Discussion also extended to the economic and environmental impacts of implementing technologies such as IoT devices and the blockchain. It was noted that these technologies could be expensive to deploy and may have significant environmental footprints [12]. For instance, blockchain technology, while enhancing data traceability, is energy-intensive and may contribute to greenhouse gas emissions. A balanced approach that considers the economic and environmental sustainability of these technologies is necessary.

Some recommendations are as follows:

- Implementing Blockchain Technology: We should use the blockchain to monitor and trace data usage, ensuring transparency and fairness in data management. Alternative technologies such as distributed ledger systems, federated learning approaches, and permissioned blockchains were also briefly considered during our discussions, including IBM food trust, TraceXTech, Spydra, among others. However, the blockchain was highlighted for its established ability to foster trust and transparency among diverse stakeholders in the dairy industry;
- Ensuring Secure IoT Devices: we should develop standards to ensure that IoT devices used in dairy farming are secure and that the data collected are used ethically;
- Promoting Sustainable Practices: we should adopt sustainable approaches to data generation, distribution, and utility, focusing on minimizing environmental impacts;
- Fostering Equitable Value Distribution: we should ensure that all stakeholders, especially farmers, share the benefits of technological advancements equitably.

#### 3.2.8. Global Compliance and Standards

Differences in data protection regulations across various regions pose challenges for establishing uniform data management practices. A collaborative approach is needed to develop global compliance standards that protect data integrity and ensure fair use. The cooperative nature of the dairy industry in Canada, where data management practices are more standardized and industry-driven, was highlighted. In contrast, the fragmented nature of the dairy industry in the USA, with varying regulations and standards, presents additional challenges for developing uniform data management practices. In India, the landscape of data management in dairy farming is characterized by significant variability in farm sizes and levels of technological adoption. While large farms are increasingly adopting digital technologies for data collection, small farms often rely on traditional methods. The lack of standardized legal frameworks for data protection and the need for greater transparency and farmer awareness were emphasized.

Global standards must consider not only the legal frameworks for data ownership but also the operational policies that allow producers to opt out while maintaining a fair balance of costs and benefits. Policies should explore mechanisms like differentiated pricing for services and tools, ensuring equitable value distribution without penalizing non-participating producers disproportionately.

The healthcare and financial service industries provide valuable examples of how data ownership and privacy challenges can be addressed through well-defined policies and frameworks. In healthcare, policies such as HIPAA (Health Insurance Portability and Accountability Act) in the USA emphasize patient ownership of medical data while allowing for their secure sharing under strict regulations. This approach ensures both data privacy and their utility for broader purposes, such as medical research and innovation. Similarly, in financial services, data-sharing agreements are governed by stringent rules that balance consumer privacy with the operational needs of institutions. These sectors highlight the importance of transparency, explicit consent, and equitable value distribution—principles that are equally relevant to dairy farming. For example, adopting frameworks inspired by healthcare data policies could empower farmers to retain ownership of their data while enabling the data’s use for industry-wide benefits, such as genetic evaluations and productivity benchmarking. Likewise, implementing secure data-sharing platforms, akin to those in financial services, could facilitate trust and collaboration among stakeholders. By adapting these proven approaches to the unique needs of the dairy industry, stakeholders can address concerns about data misuse and inequity while fostering innovation and sustainability.

Addressing these challenges involves not only developing comprehensive guidelines but also ensuring that they are effectively communicated and implemented across different regions. Collaboration among international bodies, governments, and industry stakeholders is crucial for creating robust, harmonized standards. By promoting transparency and equitable data practices, the dairy industry can better protect the rights of farmers while fostering innovation and sustainability.

Some recommendations are as follows:

- Develop Harmonized Global Standards: we should establish and promote international guidelines for data protection and privacy to address regional discrepancies and ensure uniform practices across the dairy industry;
- Foster Collaboration: we should encourage cooperation among international bodies, governments, and industry stakeholders to develop and implement these standards effectively;
- Enhance Transparency: we should promote transparent data management practices and ensure explicit permissions are obtained for data use;
- Support Farmer Awareness: we should increase efforts to educate farmers on data rights and the implications of data sharing to enhance their understanding and control over their data;
- Implement Robust Data Protection Measures: we should establish strong data protection protocols to safeguard data integrity and prevent unauthorized access or misuse.

By focusing on these areas, the dairy industry can navigate the complexities of global data management, ensuring that all stakeholders benefit from technological advancements while maintaining ethical and sustainable practices.

## 4. Conclusions

The group discussions underscore the critical importance of establishing clear guidelines and ethical frameworks for data ownership and privacy within the dairy industry. Addressing these multifaceted issues requires a collaborative approach that includes technological advancements, stakeholder engagement, and the development of global standards. Ensuring equitable value distribution is paramount; the benefits derived from data must be shared fairly among all stakeholders, particularly the farmers who generate the data. Transparent data management practices are essential, with an emphasis on obtaining explicit permissions for data use and maintaining open communication about how data are utilized. The development and harmonization of international guidelines for data protection and privacy are crucial to facilitate uniform practices across different regions and to address discrepancies in existing regulations such as GDPR in Europe and varying laws in countries like India and the USA.

Moreover, the integration of advanced technologies like IoT devices and the blockchain offers both opportunities and challenges, enhancing data tracking and transparency while raising questions about data security and farmer consent. A balanced approach that considers the economic and environmental sustainability of these technologies is necessary. Implementing robust data protection measures and promoting sustainable data generation, distribution, and utility practices are vital to safeguarding data integrity and ensuring ethical usage.

By embracing innovations such as IoT, the blockchain, and data spaces, the dairy industry can build a robust infrastructure for secure, equitable, and sustainable data utilization. These technologies offer practical pathways for monetizing data and fostering collaborative environments that benefit all stakeholders.

By fostering an environment of transparency and fairness, the dairy industry can harness the power of data to drive innovation and sustainability. Looking ahead, the future of data ownership in the dairy industry will be shaped by emerging technologies such as AI and edge computing. These technologies promise to revolutionize how data are managed by enabling real-time processing and decision-making directly at the source, reducing reliance on centralized systems. However, their adoption also presents challenges related to data security, privacy, and equitable access. Addressing these issues will require the dairy sector to engage in proactive policy development, ensuring that these innovations are implemented in ways that promote fairness, transparency, and sustainability. By thoughtfully integrating these advancements, the industry can create a more resilient and adaptive data ecosystem that empowers all stakeholders while safeguarding their interests. This comprehensive approach will not only protect the rights of data generators but also enhance the overall efficiency and productivity of the industry, as evidenced by the results emphasizing the role of transparency, robust data protection measures, and technological advancements. These practices collectively pave the way for a more secure, trustable, and equitable data management ecosystem.

To further the conversation on data ownership, privacy, and management, organizations like the International Committee for Animal Recording (ICAR) and AgGateway are critical. Their contributions to global standardization, data interoperability, and stakeholder collaboration provide essential support for addressing the challenges highlighted in this paper.

Realistically implementing the proposed guidelines requires a focus on cost-effective, farmer-centered solutions that account for the resource constraints and priorities of farmers. Standard-setting organizations like ICAR and AgGateway can play pivotal roles by providing frameworks, certification processes, and tools that reduce the burden of compliance on individual farmers while ensuring consistency and interoperability across the sector.

To promote adoption, implementation strategies could include subsidized technology programs, farmer education, and training initiatives, as well as incentives for participation in collaborative data-sharing systems. Engaging cooperatives and producer organizations as intermediaries can further enhance farmer involvement by reducing individual costs and amplifying collective bargaining power. By combining these practical measures with strong governance and robust stakeholder engagement, the dairy sector can build a scalable, sustainable infrastructure for equitable data management and privacy.

While the topic of data ownership is broad and conceptual, introducing specific cases or models for debate can help the industry navigate these issues pragmatically. Concrete examples, such as differentiated pricing models or opt-out options, can guide discussions and inform the development of equitable policies that balance collective innovation with individual rights.

The conclusions presented in this paper reflect a consensus among the group for key recommendations, such as the necessity of transparent data practices and equitable value distribution. However, certain recommendations, such as global compliance standards, were met with diverse opinions, indicating a need for further exploration and industry-wide discussions. While this paper raises awareness of the challenges and proposes general actions, it does not claim to solve these complex issues but rather aims to serve as a pathway to guide future efforts in the dairy industry.

## Data Availability

Not applicable. This commentary paper does not involve the generation or use of any datasets. It is based solely on the opinions, expertise, and discussions of the authors.
